# Detection of Circulating Tumor Plasma Cells in Monoclonal Gammopathies: Methods, Pathogenic Role, and Clinical Implications

**DOI:** 10.3390/cancers12061499

**Published:** 2020-06-08

**Authors:** Luzalba Sanoja-Flores, Juan Flores-Montero, Martín Pérez-Andrés, Noemí Puig, Alberto Orfao

**Affiliations:** 1 Translational and Clinical Research Program, Centro de Investigación del Cáncer and Instituto de Biología Molecular y Celular del Cáncer, Consejo Superior de Investigaciones Científicas (CSIC)- University of Salamanca, 37007 Salamanca, Spain; lucysanoja@usal.es (L.S.-F.); jflores@usal.es (J.F.-M.); mmmar@usal.es (M.P.-A.); 2 Centro de Investigación Biomédica en Red de Cáncer, CIBER-ONC number CB16/12/00400, Instituto Carlos III, 28029 Madrid, Spain; 3 Department of Hematology, University Hospital of Salamanca, IBSAL, IBMCC (USAL-CSIC), 37007 Salamanca, Spain; npuig@saludcastillayleon.es; 4 Centro de Investigación Biomédica en Red de Cáncer, CIBER-ONC number CB16/12/00233, Instituto Carlos III, 28029 Madrid, Spain

**Keywords:** circulating tumor plasma cells, monoclonal gammopathy of undetermined significance, multiple myeloma

## Abstract

Cancer dissemination and distant metastasis most frequently require the release of tumor cells into the blood circulation, both in solid tumors and most hematological malignancies, including plasma cell neoplasms. However, detection of blood circulating tumor cells in solid tumors and some hematological malignancies, such as the majority of mature/peripheral B-cell lymphomas and monoclonal gammopathies, has long been a challenge due to their very low frequency. In recent years, the availability of highly-sensitive and standardized methods for the detection of circulating tumor plasma cells (CTPC) in monoclonal gammopathies, e.g., next-generation flow cytometry (NGF), demonstrated the systematic presence of CTPC in blood in virtually every smoldering (SMM) and symptomatic multiple myeloma (MM) patient studied at diagnosis, and in the majority of patients with newly-diagnosed monoclonal gammopathies of undetermined significance (MGUS). These methods set the basis for further detailed characterization of CTPC vs. their bone marrow counterpart in monoclonal gammopathies, to investigate their role in the biology of the disease, and to confirm their strong impact on patient outcome when measured both at diagnosis and after initiating therapy. Here, we review the currently available techniques for the detection of CTPC, and determine their biological features, physiopathological role and clinical significance in patients diagnosed with distinct diagnostic categories of plasma cell neoplasms.

## 1. Introduction

Plasma cell neoplasms are an heterogenous group of end-stage antibody-producing B-cell (i.e., plasma cell) disorders [1,2]. They are characterized by an expansion of tumor plasma cells (PC), typically inside bone marrow (BM) [3], with or without involvement of peripheral blood and/or other (extramedullary) tissues such as bone, soft tissues or skin [4]. In most monoclonal gammopathy patients, tumor PC produce and release an abnormal protein (i.e., monoclonal component) that is detectable in the patient’s blood and/or urine. Monoclonal gammopathy of undetermined significance (MGUS) is the most common plasma cell neoplasm [5] and affects 3.2% of adults >50 years, and 5.3% >70 years [6]. Despite the fact that most MGUS cases will never undergo malignant transformation, previous studies have shown that MGUS is a precursor stage of multiple myeloma (MM) [7,8]. In line with this observation, malignant transformation of MGUS occurs in around 1% of patients per year [7,8]. This transformation is characterized by an increase in BM PC infiltration (≥10% BM PC) and serum monoclonal protein (≥30 g/L), and it may present in the absence of clinical symptoms (i.e., smoldering MM (SMM) [9] or as symptomatic MM [10] with evidence of underlying organ dysfunction and/or predominant BM involvement. However, in a few MM patients, extramedullary tissue lesions with limited BM infiltration occurs (i.e., macrofocal MM) [11,12]. Similarly, solitary plasmacytoma is a localized plasma cell neoplasm in which tumor PC are confined to extramedullary sites (i.e., bone or extraosseous) [13]. MM patients might also evolve to the most aggressive plasma cell neoplasm subtype, known as plasma cell leukemia (PCL), which is characterized by massive blood involvement (>2 × 10^9^ circulating tumor cells/L) [14]. In some cases, the monoclonal (most frequently lambda) light chain protein might deposit in distinct tissues and organs, affecting their function and giving rise to the so-called light chain amyloidosis, even in the presence of low numbers of tumor PC in BM and other tissues [15].

Circulating tumor plasma cells (CTPC) have long been detected in the blood of PCL patients [16,17,18], as well as in a significant fraction of MM [19,20,21,22], and to a lesser extent in MGUS cases [21,23]. From a clinical point of view, the presence and number of CTPC has been proven to have both diagnostic and prognostic implications in MGUS [21,23], SMM [24,25,26] and in symptomatic MM [20,21,27,28,29,30,31] patients. In addition, detection of CTPC has proven useful for (closer) minimally-invasive monitoring of MM after therapy [32,33,34,35]. Despite this, the reported frequency of MM and MGUS cases with detectable CTPC varies significantly, depending on the specific methodology used and its sensitivity and specificity. Thereby, usage of highly-sensitive and standardized techniques for the detection and quantitation of CTPC becomes critically important [21,35]. Here, we provide a detailed review of the currently available techniques for the detection of CTPC in patients with plasma cell neoplasms, their biological features, pathogenic role, and clinical relevance, with special focus on MGUS and MM patients. This is preceded by a brief overview of normal PC development. To this end, we performed a comprehensive literature search of PubMed and Scopus databases for indexed, English language written scientific articles published between 1974 and March 2020, which contained the following keywords: plasma cells, tumor cells, circulating and/or peripheral blood; and plasma cell neoplasms, myeloma, monoclonal gammopathy, smoldering myeloma, multiple myeloma and/or plasma cell leukemia. From all papers identified, relevant publications were reviewed and critically selected.

## 2. Normal Plasma Cell Development and Plasma Cell-Associated Phenotypes in Blood

Plasma cells represent the most advanced stage of maturation of B-cells [36,37]. Thus, PC derive from naïve and memory B-cells after they have encountered their B-cell receptor-specific antigen in secondary lymphoid tissues, where they underwent somatic hypermutation, with or without immunoglobulin (Ig) class switching [37,38]. Recently produced plasmablasts leave the secondary lymphoid tissues via the blood and migrate to survival niches, mainly located in BM [39,40] and other secondary lymphoid tissues, such as the lamina propria of the gastrointestinal tract and inflammatory tissues [41,42,43,44,45]. At such niches, recently produced plasmablasts and PC become long-lived antibody-secreting PC [46,47], or they undergo (CD95-mediated) apoptosis [39].

**Figure 1:**
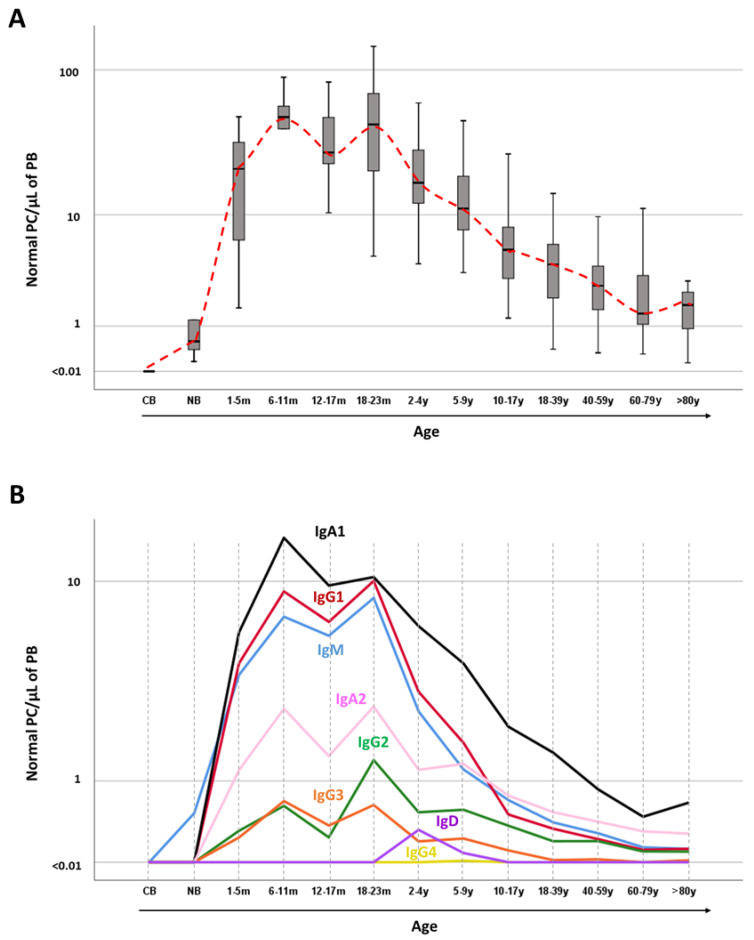
Normal plasma cell kinetics in blood from healthy individuals through life. (**A**) displays the overall distribution of total absolute normal PC counts in cord blood and peripheral blood per age group, while in (**B**) the median absolute counts of PC expressing distinct immunoglobulin isotypes and immunoglobulin subclasses are shown for the same age intervals. CB, cord blood; m, months; NB, newborn; PB, peripheral blood; PC, plasma cells; y, years. Data adapted from Blanco et al. [48].

Even when highly-sensitive techniques are used, normal circulating PC are undetectable at birth in cord blood. However, their number in blood raises exponentially during the first weeks to months of life due to continuous contact with new antigens at exposed tissues (such as at the respiratory and the gastrointestinal tracts), reaching their maximum levels at between 1 to 2 years of age. Thereafter, the number of circulating normal PC in blood continuously decreases throughout adulthood (Figure 1A). Despite this overall profile of normal PC kinetics in blood, different patterns are observed depending on the specific Ig-isotype and Ig-subclass. Thus, maximum numbers of normal IgM^+^ PC are seen first with a peak at between 6–18 months of life, when also Ig-switched normal PC expressing IgG3^+^, IgG1^+^ and IgA1^+^ peak. In turn, normal switched IgG2^+^, IgG4^+^ and IgA2^+^ PC show maximum numbers in blood later, at between 1 and 2 years of life (Figure 1B) [48]. Even when highly-sensitive techniques are used, normal circulating PC are undetectable at birth in cord blood. However, their number in blood raises exponentially during the first weeks to months of life due to continuous contact with new antigens at exposed tissues (such as at the respiratory and the gastrointestinal tracts), reaching their maximum levels at between 1 to 2 years of age. Thereafter, the number of circulating normal PC in blood continuously decreases throughout adulthood (Figure 1A). Despite this overall profile of normal PC kinetics in blood, different patterns are observed depending on the specific Ig-isotype and Ig-subclass. Thus, maximum numbers of normal IgM^+^ PC are seen first with a peak at between 6–18 months of life, when also Ig-switched normal PC expressing IgG3^+^, IgG1^+^ and IgA1^+^ peak. In turn, normal switched IgG2^+^, IgG4^+^ and IgA2^+^ PC show maximum numbers in blood later, at between 1 and 2 years of life (Figure 1B) [48].

From the phenotypic point of view, circulating normal PC show a heterogeneous profile reflecting an ongoing (continuous) maturation, with transition from recently produced tissue plasmablasts to end-stage long-lived BM PC [39]. This maturation is associated with significantly increased expression of the BLIMP-1 [47] and XBP1 transcription factors [49], which are required to suppress other transcription factors involved in the earlier stages of B-cell development (such as PAX-5 and BCL-6), and for sequential maturation of activated (e.g., germinal center) B-lymphocytes toward short-lived plasmablasts and long-lived Ig-secreting PC [50]. Thus, normal PC recently released into the blood show progressive acquisition of PC-associated markers such as CD138, the Vs38c endoplasmic reticulum-associated protein, together with (already strong) positivity for CD38 [39], progressively increased expression of the Ki67 proliferation marker [36], and the presence of cytoplasmic Igs (at lower levels than found in BM PC) [51]. At the same time, normal circulating PC show progressive loss of pan-B-cell associated markers (displaying a CD19^lo^, CD20^−/+^, HLA-DR^−/+^, CD45^lo^ phenotype) [21,39,52,53] while retaining expression of the CD81 tetraspanin adhesion molecule and the (post germinal-center) CD27 molecule. In contrast, they are constantly negative for CD117, CD56 and CD200 [52,53]. When compared to both pre- and post-germinal center B-lymphocytes, circulating normal PC also show a distinct pattern of expression of: (i) adhesion molecules, with e.g., lower CD11a levels and increased CD49d and CD31 expression; and (ii) chemokine receptors, as translated by e.g., reduced levels of CXCR5 (CD185) and CCR7 (CD197), and upregulated expression of CXCR4 (CD184) at levels intermediate between those of early tonsil plasmablasts and more mature BM PC [44,46,53]. A more extensive description of the immunophenotypic profile of circulating normal PC vs. earlier (lymphoid tissue-derived) plasmablasts and end-stage BM PC is shown in Table 1.

From a functional point of view, circulating normal PC are systematically present in the blood of healthy individuals at levels that range between 0.16–144 cells/µL in childhood and between 0.14–27.5 normal PC/µL in adults, with slight differences between younger (<50 years) and older (≥50 years) adults and individuals aged >80 years (percentile 5–95% reference ranges for Caucasians of 0.8–22 normal PC/µL, 0.3–9.7 normal PC/µL, and 0.15–17.5 normal PC/µL, respectively [48]). The overall number of circulating normal PC at different ages reflects the daily production of PC through life due to continuous triggering of B-cell responses at lymphoid tissues by our microbiome and microenvironmental antigens. Therefore, complete absence (or significantly decreased) numbers of circulating normal PC in blood after birth has been predominantly associated with profound antibody deficiency, as observed in common variable immunodeficiency, and Ig-isotype and Ig-subclass deficiencies [108]. Thereby, blood analysis of circulating normal PC provides insight into the kinetics of ongoing B-cell responses throughout the body and the potential for maintaining long-term antibody production via tissue migration and differentiation to more mature long-lived PC [53], particularly in the BM [41,109].

## 3. Detection of Circulating Tumor Plasma Cells

**Figure 2:**
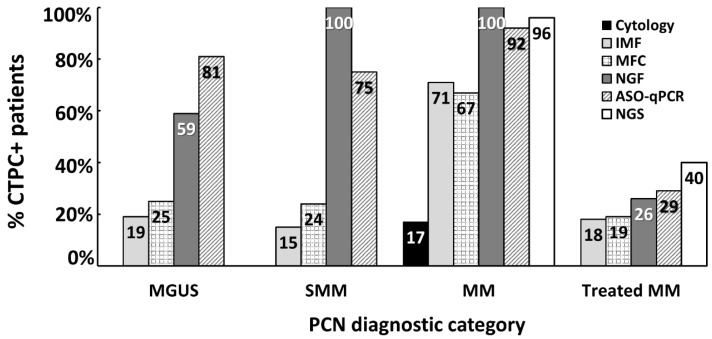
Frequency of newly-diagnosed PCN and treated MM patients with detectable CTPC in blood as assessed by distinct methods. ASO-qPCR, allele-specific oligonucleotide quantitative real-time polymerase chain reaction; CTPC, circulating tumor plasma cells; IMF, immunofluorescence microscopy; MFC, multiparameter flow cytometry; MM, multiple myeloma; NGS, next generation sequencing; MGUS, monoclonal gammopathy of undetermined significance; NGF, next generation flow; PCN, plasma cell neoplasm; SMM, smoldering MM. Data summarized from the following references in the literature [19,21,22,23,25,26,30,35,113,114,115,116,117].

During recent decades, different methods have been developed and used for the detection of CTPC (Graphical abstract [19,21,22,23,30,35] and Figure 2). Conventional cytology was first used (in combination with complete blood counts obtained in an automated hematology analyzer) for the identification of circulating PC in blood smears of patients diagnosed with monoclonal gammopathy. These counts already proved critical for the differential diagnosis between MM and PCL [18,110]. In addition, they confirmed the presence of variable PC counts in a minor fraction (17%) of all MM patients [30], which (frequently) cannot be accurately discriminated from normal/reactive plasmablasts [111] due to both the limited number of nucleated cells evaluated (i.e., <500 cells) and their morphological similarities [112], particularly among patients that show low numbers of circulating PC. Because of these limitations and the clear clinical utility of CTPC detection and quantitation in blood, conventional immunocytochemistry-based approaches were subsequently adopted. The latter technique allowed for (more specific) assessment of greater numbers of clonal PC in the blood of MM and MGUS patients based on restricted light chain expression by tumor PC [19,23]. In order to further increase the sensitivity and specificity of the above techniques, several different conventional flow cytometry [20,22,31] and next generation flow cytometry (NGF) procedures [21,35], together with polymerase chain reaction (PCR)-based, e.g., allele-specific oligonucleotide (ASO) quantitative PCR (qPCR) [19,113] and next generation sequencing (NGS) [114] techniques, were subsequently developed and tested. In addition to differences in the sensitivity reported for the above flow vs. molecular techniques, the results obtained so far are also influenced by the type of material analyzed (e.g., inclusion of nucleic acid from non-viable cells in molecular techniques) and/or the way patients were selected for analysis (e.g., inclusion of patients that reached no response or partial response together with complete response cases). The specific advantages and limitations of each of these techniques, together with their most relevant features are listed in Table 2 and described below in more detail.

### 3.1. Circulating Tumor Plasma Cell Detection in Blood Smears by Conventional Cytology

Conventional cytology is a simple, fast and inexpensive approach for (expert-based subjective) identification of CTPC with a sensitivity of ≥1% (i.e., 10^−2^) of all nucleated cells in blood, which is available at virtually every clinical diagnostics laboratory worldwide [18,30] (Table 2). The presence of CTPC by cytomorphology has long been associated with increased PC proliferation and more aggressive disease [18], which is observed (per definition) in PCL and in a small fraction of MM cases that present with high tumor load (≥5% of CTPC) and show a significantly poorer outcome -median overall survival (OS) rates of 1.1 years vs. 4.1 years for other MM cases with <5% or undetectable levels of CTPC at diagnosis, respectively [30,110] (Table 3). Thus, conventional cytomorphology remains the basis for the diagnosis of PCL [30,110]. In addition, it is of great clinical utility for the identification of MM patients that show ≥2% CTPC by Wright–Giemsa cytology at diagnosis (14.1% of untreated MM patients), who (compared to MM patients with undetected CTPC in blood) display a poorer outcome both in terms of progression free survival (PFS) (median PFS of 17 months vs. 24 months, respectively) and OS rates (median OS of 25 months vs. 45 months, respectively) [29]. Altogether, these results indicate that conventional cytology is an easy and fast approach for the detection of (high numbers) of CTPC in the blood of MM patients, particularly in cases presenting with PCL-like laboratory findings (e.g., leukocytosis and elevated serum levels of lactate dehydrogenase) and in PCL patients [18]. In contrast, conventional cytology is less useful among MGUS and SMM patients who usually present with low CTPC counts in blood. In fact, the absence of CTPC by cytomorphology should be interpreted with caution because of the limited sensitivity of the technique (Table 2).

### 3.2. Fluorescence Microscopy

For decades now, fluorescence microscopy-based analysis of immuno-stained blood-derived mononuclear cells has been recurrently applied for the detection of CTPC in the blood of MGUS and MM patients, based on Ig light-chain restriction on tumor vs. normal PC [19,24]. Briefly, this approach is based on the evaluation of anti-human light chain immunofluorescence staining patterns of density gradient isolated mononuclear cells from blood by fluorescence microscopy [25]. Overall, this technique improves (by more than one log) the sensitivity of conventional cytology with the ability to detect one clonal PC among 10,000 mononuclear cells (sensitivity of 10^−4^) [27] (Table 2). From a clinical point of view, the presence of CTPC by fluorescence microscopy is associated with disseminated disease [120], which is found in 19% [23] to 29% [19] of MGUS cases, 25% [24] to 50% [25] of SMM patients and in 71% of untreated MM cases [19] according to the literature. From a prognostic perspective, MM patients presenting with ≥4% CTPC in blood show significantly shorter median survival rates (2.4 vs. 4.5 years for MM patients with lower or undetected CTPC) [27] (Table 3). In addition, the presence of CTPC in the blood of SMM patients has also been associated with shorter time to progression (TTP) rates compared to CTPC-negative SMM patients (median TTP of 9 vs. 30 months, respectively) [24]. This is even more evident among SMM cases presenting with higher CTPC counts (>5000 × 10^6^ CTPC/L or >5% cytoplasmic Ig-positive CTPC/mononuclear cells) who show median time to progression rates of 12 vs. 57 months for other SMM patients with undetected or lower CTPC numbers [25]. Similarly, MGUS patients that have CTPC by fluorescence microscopy also show a more adverse prognosis vs. CTPC-negative MGUS patients (median progression free survival of 138 months vs. not reached, respectively) [23]. Of note, recent CTPC detection techniques based on specific pre-analytical PC-enrichment procedures such as the CELLSEARCH^®^ platform developed by Menarini-Silicon Biosystems (Castel Maggiore, Italy), have proven to increase the frequency of patients that show CTPC in blood to >85% of all MGUS, SMM and MM cases studied at diagnosis [121]. In addition, preliminary results suggest that this latter technology might also be of potential clinical utility among treated MM patients who reach complete response, because those patients that had higher CTPC counts in blood (≥100 CTPC/4 mL or ≥25 CTPC/mL) displayed a more adverse prognosis compared to patients with lower numbers of CTPC [121].

Despite the clinically relevant information provided by fluorescence microscopy (and other imaging-based approaches), this technique still has several important limitations that hamper its routine use in many laboratories [20,122]. These mainly include: (i) the relatively limited sensitivity reached, (ii) the fact that fluorescence microscopy is a laborious and time-consuming technique, (iii) the need for a pre-enrichment step to isolate mononuclear cells with high potential for uncontrolled (selective) cell loss that might specifically affect CTPC at the same time it discards potentially relevant information on residual (non-mononuclear) hematologic blood cells [123], and (iv) the need for a fluorescence microscope (or more complex instrumentation) usually not available for routine diagnostics in many haemato-oncology laboratories (Table 2).

### 3.3. Conventional Multiparameter Flow Cytometry

Flow cytometry has long been recognized as a well-suited methodology for the enumeration of CTPC in blood of plasma cell neoplasms patients [20,22,122]. This technique is an easy, fast (<4 h), affordable and worldwide available approach which has been extensively used to identify, characterize and count CTPC in the blood of patients with plasma cell neoplasms. However, the lack of standardized protocols (i.e., very heterogeneous antibody panels have been used and variable numbers of cells analyzed per sample), the highly variable sensitivity levels reached for detection of CTPC (which translate into variable frequencies of CTPC-positive patients), together with the need for fresh (<24–48 h) samples, have limited the reproducibility of results, and thereby, its broader use and applicability (Table 2). Despite these limitations, flow cytometry has shown that the presence and number of CTPC in blood (both at diagnosis and after therapy) has important clinical implications in MM, and to a lesser extent, also in MGUS, and SMM patients [20,22,124].

Early multiparameter flow cytometry studies in MGUS based on very limited numbers of markers -e.g., three-color antibody combinations of CD45, CD38, and cytoplasmic (cy)Igκ or cyIgλ identified CTPC in the blood of 25% of cases with a median (range) of 0.3% (0.06–0.97%)- CTPC from blood-derived mononuclear cells [19]. However, these results could not be confirmed in subsequent studies using more sensitive approaches based on seven-color flow cytometry and analysis of ≥10^6^ cells that reported counts of <0.0035% of CTPC in the blood of the majority of MGUS patients (93%) at diagnosis [124].

Similarly, in the few SMM-based flow cytometry studies reported in the literature, remarkably different frequencies of CTPC in blood were observed depending on the specific approach used. Thus, in an early three-color flow cytometry study in a small cohort of SMM patients, CTPC were identified in 3/8 cases (37.5%) [19]. Conversely, a more recent report on a larger series of 100 SMM patients studied at diagnosis using six-color cytometry based on staining of approximately 150,000 mononuclear cells for CD45, CD19, CD38, CD138, cyIgκ and cyIgλ, identified CTPC in blood of only 24% of the patients [26]. Of note, the detection of CTPC by multiparameter flow cytometry predicted shorter time to progression rates from SMM to MM (median TTP of 10 months vs. not reached for CTPC-positive vs. CTPC-negative patients) [26].

In contrast to MGUS and SMM, more studies have investigated the frequency and clinical implications of CTPC in the blood of MM patients by multiparameter flow cytometry, both at diagnosis and after starting therapy. Thus, CTPC have been identified by multiparameter flow cytometry in 50% to 75% of newly-diagnosed MM cases [19,20,22,28,31], depending on the number of markers and the specific antibody panels used [19,22,28,31], the number of cells analyzed [22,28,31], and the sample preparation protocol [19,22,28,31]. Importantly, these studies showed that the rate of CTPC-positive cases in blood among untreated MM patients increases approximately 1.4 fold from density gradient mononuclear cell isolation-based approaches [22,28] to whole blood flow cytometry protocols [31], potentially due to specific loss of CTPC during mononuclear cell isolation procedures. Additionally, in these studies, absolute CTPC counts by multiparameter flow cytometry in the blood of MM patients measured at diagnosis varied between 2.5–3 CTPC/µL [22,31].

From a prognostic point of view, higher CTPC counts in blood as detected by multiparameter flow cytometry (regardless of the specific threshold proposed) are systematically associated with an independent adverse prognostic impact among newly-diagnosed MM [22,124]. Thus, the presence of ≥0.0035% (vs. <0.0035%) CTPC in the blood of untreated MM patients translated into a worse outcome, with lower three-year time to progression (65% vs. 34%) and overall survival (52% vs. 90%) rates, respectively, independently of the therapeutic regimen used or the presence of adverse cytogenetics as defined by the International Myeloma Working Group criteria -i.e., Revised International Staging System (R-ISS)- [124]. Similarly, inferior overall survival (OS) rates were found in MM patients with ≥400 CTPC/150,000 mononuclear cells at diagnosis (median OS of 32 months vs. not reached for cases with <400 CTPC/150,000 mononuclear cells) [28]. In line with these findings, a recent study on newly-diagnosed MM shows that the presence of ≥5 CTPC/µL of blood is associated with a significantly shorter time to next therapy (TTNT) and OS rates, compared with cases showing lower (<5 CTPC/µL) or undetected CTPC in blood (median TTNT of 21, 28 and 43 months, respectively, [22] and median OS of 46, 76 and 89 months, respectively [22]). Based on these results, the authors suggest that R-ISS I and R-ISS II MM patients presenting with ≥5 CTPC/µL in blood at diagnosis might display a similarly dismal prognosis to R-ISS III MM patients [22].

Several flow cytometry-based studies also recognized the (adverse) prognostic impact of CTPC in treated MM patients, where decreasing frequencies of CTPC are associated with a progressively better response to therapy [33,116,125,126]. Overall, six-color flow cytometry (or higher) detected CTPC in 18.7% to 19.3% of treated MM patients prior to stem cell mobilization for autologous stem cell transplantation [116,125,126]. In MM patients who reached complete response prior to stem cell mobilization for autologous stem cell transplantation, the frequency of CTPC-positive patients ranges from 0% [32,33,34] to 14% [116,125,126], depending on the specific multiparametric flow cytometry approach used (e.g., for three- [32] to six-color cytometry [34]), the number of cells analyzed [125], and the sample preparation technique (e.g., staining of mononuclear cells [116] vs. erythrocyte-lysed whole blood [32,126]). In contrast, a significantly greater frequency (approximately 64%) of CTPC-positive cases was found among relapsed MM patients at the time of stem cell collection [33]. In line with these findings, a poorer outcome (with significantly shorter time to progression, progression free survival and/or overall survival rates) of treated MM patients showing CTPC in the blood by multiparameter flow cytometry has been recurrently confirmed by several groups, independently of the therapeutic regimen administered and the depth of clinical response achieved [33,116,125,126] (Table 3).

### 3.4. Next Generation Flow Cytometry (NGF)

NGF approaches have been recently developed for minimal/measurable residual disease (MRD) monitoring in the BM of treated MM patients [127,128]. Early NGF studies already demonstrated a significantly greater sensitivity (sensitivity of ≤2 × 10^−6^) and reproducibility for NGF vs. classical (8–10-color) flow cytometry [127] (Table 2). This is mostly due to: (i) evaluation of significantly greater numbers of cells per sample (i.e., ≥10 × 10^6^ cells) achieved via ammonium chloride-based bulk-lysis of blood samples prior to antibody staining; (ii) an optimized two eight-color tube antibody combination; and (iii) usage of computer-assisted software tools for more objective and reproducible automated data analysis (i.e., the INFINICYT software from Cytognos Sl, Salamanca, Spain) [127].

More recently, NGF has also been applied for the detection of CTPC in the blood of MGUS, solitary plasmacytoma, SMM and MM (including a small group of macrofocal MM) patients [21]. In this latter study, NGF showed that the presence of CTPC in blood is a sign of systemic disease with significantly lower rates of CTPC among patients with localized vs. disseminated diagnostic subtypes of monoclonal gammopathy: ≤25% in solitary plasmacytoma and macrofocal MM cases vs. 59% in MGUS and 100% in both SMM and MM patients [21]. Overall, these are unprecedently high frequencies of CTPC-positive cases vs. those previously reported using other (less sensitive, i.e., flow) approaches [19,20,22,26,31]. More interestingly, the number of CTPC in blood progressively increased from MGUS (median CTPC count of 0.008 CTPC/μL) to SMM (median of 0.16 CTPC/μL) and MM (median of 1.9 CTPC/μL) patients. Noteworthy, a cutoff of 0.058 CTPC/μL was able to discriminate MGUS from MM patients with high accuracy (80% sensitivity and 80% specificity) [21].

From a prognostic point of view, the number of CTPC detected by NGF also proved to efficiently discriminate between MGUS cases with high (≥0.058 CTPC/μL) vs. low (<0.058 CTPC/μL) risk of progression to MM (median time to progression of 31 months vs. not reached, respectively), and to identify newly-diagnosed MM patients with reduced (≥0.1 CTPC/μL) vs. prolonged (<0.1 CTPC/μL) progression free survival (PFS) and overall survival (OS) (median PFS of 22 months vs. not reached and 75% OS of 17 months vs. not reached, respectively). Interestingly, MM patients who had CTPC counts at diagnosis similar to those of MGUS (<0.1 CTPC/μL) displayed a significantly favorable long-term outcome, independent of their response to therapy (e.g., complete response and BM MRD status). In contrast, in this study, the number of CTPC found in blood by NGF did not show a significant impact on the outcome of SMM patients, probably due to the limited of number of cases analyzed [21] (Table 3).

Recently, the presence of CTPC in the blood of MM patients has also been evaluated by NGF after starting therapy. Thus, results in a large cohort of 137 real-world MM patients treated outside of clinical trials demonstrated CTPC by NGF in 26% of treated MM cases, including 17% of cases who had achieved complete response/stringent complete response [35], a significantly greater percentage than previously shown by conventional multiparameter flow cytometry approaches [34,116,125,126]. As expected, all treated MM patients who had CTPC in the blood by NGF were also MRD-positive in the BM, while the remaining two-thirds of BM MRD-positive cases did not show blood involvement by CTPC. Despite the lower sensitivity of detection of CTPC in blood vs. BM MRD, persistence of CTPC after treatment by NGF was associated with five-fold reduced progression free survival rates compared to CTPC-negative patients (median PFS of 9 vs. 46 months, respectively), independent of the patient’s tumor cytogenetics, the response achieved (complete response/stringent complete response vs. non-complete response) including the BM MRD status, and the treatment phase at which the presence of CTPC in blood had been assessed [35]. Interestingly, sequential blood CTPC monitoring by NGF predicted better long-term outcomes than single time-point assessments. Thus, treated MM patients who were persistently CTPC-negative in blood (i.e., CTPC −/−) or turned negative after a first positive result (i.e., CTPC +/−), had significantly superior progression free survival rates at two years (92.5%) compared to cases with positivity for CTPC in the last NGF analyses (CTPC −/+ or CTPC +/+ cases) (41%) [35].

Altogether, these findings demonstrate that NGF is a highly-sensitive technique that allows identification and quantitation of CTPC in blood at diagnosis in the majority of MGUS cases and virtually all SMM and MM cases [21]. In addition, it provides valuable prognostic information in both patient groups and represents a new (minimally invasive) surrogate biomarker for BM MRD-positivity among treated MM patients [35].

### 3.5. Molecular (ASO-qPCR and Next Generation Sequencing) Techniques

Although molecular techniques cannot strictly detect entire (tumor) cells, including CTPC, quantitation of some unique genetic (e.g., DNA) tumor markers such as patient-specific *IGH-V(D)J* gene rearrangements of CTPC has been long proven to closely reflect the tumor cell load [114,118,129]. This is in contrast with techniques that measure the M-component (e.g., immunofixation and/or mass spectrometry) [130,131], because the serum levels of the monoclonal protein produced by the tumor PC depend on several parameters other than the number of CTPC, such as the tumor load in other tissues (e.g., BM), the (highly variable) amount of protein produced by individual tumor PC among different plasma cell neoplasms patients, and its half-life [132]. So far, most studies in which molecular techniques have been used for CTPC detection in plasma cell neoplasms have focused on MM patients evaluated at diagnosis and/or after starting therapy [133]. Of note, virtually all these studies used ASO-qPCR [113,118,129], whereas a few NGS-based studies have been more recently reported [114,117].

ASO-qPCR is a highly specific and sensitive (<10^−5^ to 10^−6^) molecular method for the detection and quantification of CTPC, based on the identification and follow-up of patient-specific Ig heavy chain (*IGH*) complementary determining region 3 (CDR3) gene rearrangements in the blood of MM patients [118,129]. Thus, ASO-qPCR-based studies have shown the presence of CTPC in the blood in 67% [118] to 93% [113,129] of MM patients studied at diagnosis (the calculated number of CTPC detected ranged from 0.001% to 1.0% of all white blood cells) [129]. In turn, another preliminary ASO-qPCR-based study identified CTPC in 81% of MGUS cases, although the authors could not ensure that these patients were not in an advanced stage of the disease (with a constant increase of the monoclonal component) [19] (Figure 2). Similarly to NGF, the presence of CTPC by ASO-qPCR has been reported in the blood of between 24% of treated MM cases who are incomplete responders [113] to 73% of relapsed patients [118]. From a prognostic point of view, detection of CTPC by ASO-qPCR has been associated with impaired survival rates both in newly-diagnosed MM patients (median progression free survival of 26 months vs. 66 months for CTPC negative cases, respectively), and in MM cases studied after three months of high-dose therapy plus autologous stem cell transplantation (median progression free survival of 4 months vs. 15 months, and median overall survival of 17 months vs. 52 months for CTPC-positive vs. CTPC-negative cases, respectively) [118] (Table 3). However, data on the potential diagnostic and prognostic implications of CTPC detection by ASO-PCR in both MGUS and SMM patients is still limited and remains to be investigated [19].

As an advantage, ASO-qPCR and other (e.g., NGS) molecular techniques do not require fresh samples, since they can use stored blood-derived nucleic acids to evaluate CTPC. In contrast, they have a more limited applicability (range: 42% to 95% of cases) in MGUS, MM and other plasma cell neoplasms [133,134,135]. Additionally, these molecular PCR-based techniques require analysis of a baseline (e.g., diagnostic or relapse) sample to identify the patient-specific *IGH* rearrangement(s) [135,136] (Table 2). For this purpose, a pre-enrichment/purification of tumor PC via isolation of mononuclear cells through density gradient centrifugation or via magnetic and fluorescence activated cell sorting (FACS) of e.g., CD138^+^ PC is frequently required, even when diagnostic BM samples are available [133].

Next generation sequencing of *IGH*-V(D)J is a more sensitive (<10^−6^) and applicable (88–95%) molecular technique than ASO-qPCR [133,135,136] (Table 2). Thus, analysis of blood leukocyte DNA samples identified the clonotypic V(D)J rearrangements of CTPC by NGS in between 71% [117] and 78% [114] of MM cases at baseline (e.g., before starting therapy) and in 40% of treated MM patients (who mostly reached partial response to therapy) [117]. Nonetheless, in one study, the frequency of CTPC in blood increased to 96% of newly-diagnosed MM patients when RNA was used instead of DNA to detect tumor *IGH*-V(D)J gene rearrangements [114]. However, to date, molecular analysis by next generation sequencing of blood-derived CTPC has preferentially focused on the molecular characterization of purified CTPC in order to better understand the biology of the disease [119,121,137,138,139]. In this regard, a recent study based on single-cell RNA sequencing reported that overexpression of *CENPF* and *LGALS1* genes in CTPC from MM patients was associated with reduced progression free survival rates [119] (Table 3). In contrast, the clinical impact of next generation sequencing-based analysis of CTPC in MGUS, SMM and other plasma cell neoplasms currently remains unknown and deserves further investigation [137].

## 4. Biological Features and Physio-Pathological Role of CTPC in Plasma Cell Neoplasms

**Figure 3:**
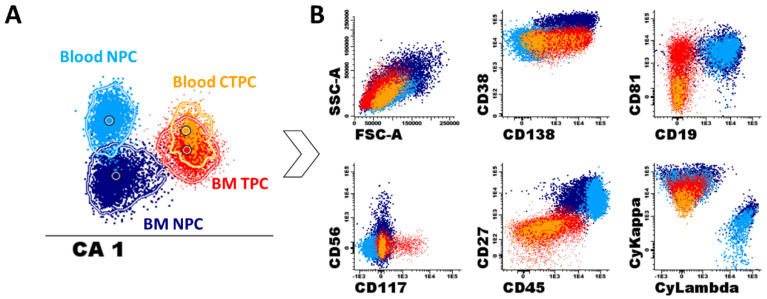
Example of the immunophenotypic differences observed by NGF between normal and tumor PC from paired BM and blood samples from a representative MM patient studied after HDT followed by ASCT. Multivariate canonical analysis (CA) plot (**A**) and classical two-dimensional dot plot representations (**B**) show the immunophenotypic differences between BM TPC (red dots) and PB CTPC (orange dots), and between normal PC from BM (dark blue color) and PB (light blue dots) following the EuroFlow NGF-MM minimal residual disease (MRD) approach. ASCT, autologous stem cell transplantation; BM, bone marrow; CTPC, circulating tumor plasma cells; HDT, high-dose therapy; MM, multiple myeloma; NPC, normal plasma cells; PB, peripheral blood; PC, plasma cells; TPC, tumor PC. Data modified from Sanoja-Flores et al. [35].

At present, the precise biological meaning and physio-pathological role of blood CTPC in MGUS, MM and other plasma cell neoplasms is not yet fully understood [23,140,141,142]. However, it is commonly accepted that CTPC reflect the biology of the tumor [20,35], representing a marker (related to, but independent of) the overall tumor burden [21,28,31,32,114]. For decades, the association observed between the presence (and greater numbers) of CTPC in blood and a higher tumor burden in the BM of MGUS, MM, PCL and other plasma cell neoplasms, has led to the notion that CTPC are a functionally unique population of BM-derived tumor PC [20,23,24]. However, more recent data indicates that CTPC usually represent a distinct subclone of BM PC [119,143,144] with a more immature phenotype (Figure 3) and quiescent profile [21,144,145]. Thus, the association between blood and BM PC numbers could not be confirmed in several studies [20,118,129], or it has been shown to be a non-linear correlation [21,32] independent of sample quality (e.g., hemodilution). Based on these findings, it has been hypothesized that CTPC might represent tumor cells that have migrated into the blood as a consequence of the influence of the tumor on its BM microenvironment, leading to escape from local immune surveillance [142] and increased levels of hypoxia [146]. At present, it is well-established than a hypoxic microenvironment stimulates the secretion of large amounts of vascular endothelial growth factor (VEGF) which locally promotes angiogenesis and tumor growth [140], together with the loss of expression of several (stroma) adhesion molecules on tumor PC [20,146], such as CD56, CD138, CD81 and CD117 [21,144]. This would favor tumor cell release and circulation. In addition, circadian variations of stromal cell derived factor 1 (SDF1) and chemokine receptor 4 (CXCR4) plasma levels [144], together with downregulation of PC-associated expression of integrins like CD11a, CD11c, CD29, CD49d and CD49e [144], and activation antigens (such as CD38 and CD27) [21,144] on CTPC vs. BM tumor PC, might also favor the release of tumor PC into the circulation.

Despite all of the above, the overall immunophenotypic profile of CTPC suggests a more immature stage of PC maturation compared to (paired) BM tumor PC (Figure 3) [21,144]. Thus, CTPC show a phenotype consistent with lower protein synthesis (as reflected by decreased levels of the endoplasmic reticulum-associated Vs38c marker and cytoplasmic Ig) [21], and reduced proliferation with both lower bromodeoxyuridine (BrdU) and deep red anthraquinone 5.5 (DRAQ5.5) uptake [144,145], and Ki67 expression levels [21]. In addition, CTPC show greater in vitro migration potential and increased in vitro self-renewing capacity for generating tumor PC colonies [144]. Altogether, these findings suggest that CTPC might represent the stem-cell like counterpart of tumor PC [21,144]. If this hypothesis holds true, higher numbers of CTPC in blood (with greater capacity of self-sustaining independently of the BM microenvironment), would potentially translate into faster and more extensive spread of the tumor via the bloodstream [25,142,147] throughout the BM and to extramedullary sites [21,142]. This would ultimately contribute to explaining, at least in part, the association observed between higher CTPC counts in blood and more advanced and aggressive features of the disease [21,22,25,28].

**Figure 4:**
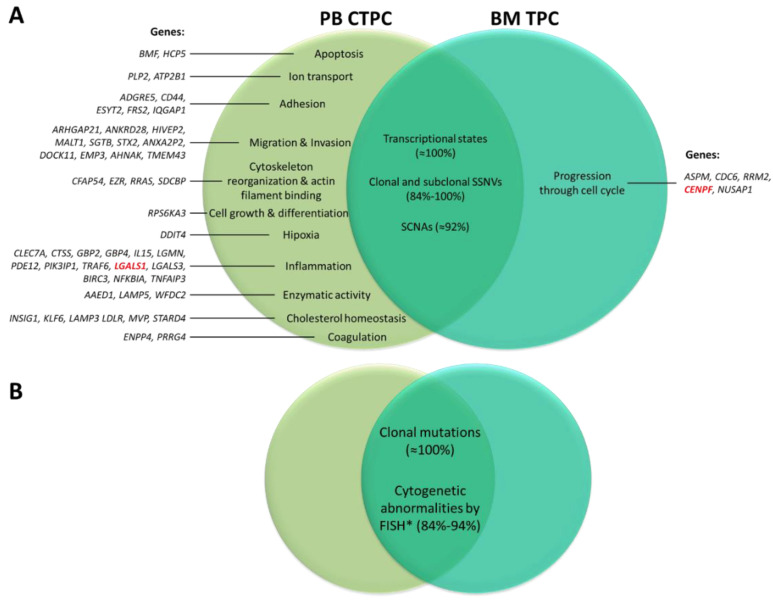
Differential gene expression profile (**A**) and genetic alterations (**B**) of blood circulating tumor plasma cells (CTPC) compared to paired bone marrow (BM) TPC in MM. The light green circle represents the most relevant genes differentially expressed in blood CTPC vs. BM TPC. In turn, the dark green circle represents genes differentially expressed in BM TPC vs. their blood counterpart. The intersection of both circles indicates the genetic profile shared between blood CTPC and BM TPC. Genes depicted in red have been reported as independent prognostic factors for progression-free survival and overall survival in MM. * Cytogenetic alterations involve del17p, t (4;14), t (14;16). Clonal mutations refer to genes such as *KRAS, NRAS, BRAF* and *TP53*. FISH, fluorescent in situ hybridization; MM, multiple myeloma; PB, peripheral blood; SCNAs, somatic copy-number alterations; SSNVs, somatic single-nucleotide variants; TPC, tumor plasma cells. Data modified from [119,121,137,138,139,143].

From a genomic point of view, several studies have shown that (purified) CTPC (mostly) from MM patients reproduce the pattern of somatic mutations present in BM tumor PC with relatively high levels of (sub)clonal heterogeneity [137,143]. Thus, CTPC show overexpression of genes (and mutations/genetic alterations) associated with drug resistance [143], the inflammatory response (e.g., *BIRC3* or *TNFAIP*3), hypoxia (e.g., *DDIT4*), cell migration (e.g., *CFAP54*, *EZR*, *EMP3* or *AHNAK*) and metastasis (e.g., *AGR2*, *DDX5*, *MALAT1*, *TMED2*, *TPT1*), together with downregulation of genes responsible for progression through the cell cycle (e.g., *CENPF* or *CDC6*) [119] (Figure 4A). In addition, the presence of CTPC in blood has also been related to ancestral cytogenetic profiles and unique cytogenetic alterations, including t(4;14) [28,29,116], deletion 13q [29], deletion 17p, t(11;14) and t(14;16) [22] (Figure 4B).

## 5. Clinical Implications of CTPC in Plasma Cell Neoplasms

Based on the above findings, at present, CTPCs are considered a sign of active [27,32] and disseminated PC disease (i.e., from MGUS to SMM and MM) [21,142], while less frequent in tissue-localized plasma cell neoplasms (e.g., solitary plasmacytoma or macrofocal MM) [21] or a subset of MM patients with an MGUS-like phenotype that predominantly show bone disease/infiltration [21,27]. In addition, higher CTPC counts in blood are currently considered an adverse prognostic marker in MGUS and both newly-diagnosed and treated MM patients independent of the method used for their detection and quantitation [21,22,23,25,35,125,126]. At diagnosis, the prognostic value of CTPC in MM is independent from patient age, cytogenetic risk and the R-ISS stage of the disease [20,124]. Similarly, persistence of CTPC in the blood of treated MM patients predicts shorter survival (e.g., lower progression free survival and overall survival rates) independent of patient age, high-risk cytogenetics, the type of therapy administered, and the response achieved, as evaluated by the serum M-component (assessed by immunofixation and the free light-chain ratio), as well as the BM MRD status [33,35,125].

Detection of CTPC by currently available highly-sensitive approaches such as NGF is a less sensitive MRD marker than BM MRD for monitoring treated MM patients [35]. However, blood monitoring of CTPC has multiple advantages compared to BM MRD. Thus, detection/quantitation of CTPC in blood is a minimally-invasive approach that, unlike BM MRD, is not affected by (patchy) disease distribution and/or hemodilution [148]. At the same time, it is well-suited for (more) frequent monitoring of newly-diagnosed MGUS and treated MM patients who attain complete response [21,32,35,118], providing information that is complementary to serum immunofixation [35], for example, for the follow-up of non-secretory PC tumors [129].

Despite all of the above, detection and monitoring of CTPC levels in the blood are not part of current clinical diagnostic and treatment response criteria, which still rely on conventional biochemical (i.e., serum/urine immunofixation, protein electrophoresis and/or free light chain ratio status), cytomorphological (e.g., blood and BM PC counts), immunophenotypic (e.g., BM MRD by NGF), molecular (e.g., next generation sequencing-based BM MRD) and imaging criteria for the detection/monitoring of e.g., bone lesions and soft tissue plasmacytomas [130].

**Figure 5:**
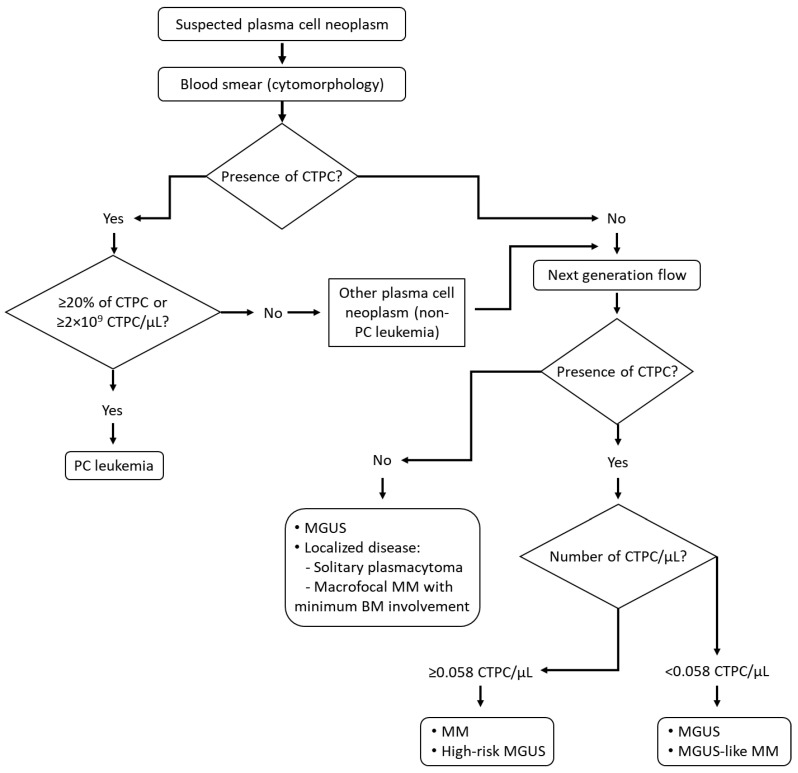
Proposed strategy for a comprehensive diagnostic work-up of patients suspected of or diagnosed with monoclonal gammopathy based on the assessment of circulating tumor plasma cells by complementary technologies. Please note that the proposed approach should be used in combination with currently established diagnostic approaches. BM, bone marrow; CTPC, circulating tumor plasma cells; MGUS, monoclonal gammopathy of undetermined significance; MM, multiple myeloma; PC, plasma cell.

Based on the above findings, here we propose a diagnostic algorithm to assess the presence of CTPC in the diagnostic work up of patients suspected of plasma cell neoplasms that might contribute to a greater diagnostic efficiency and closer monitoring of the patients (Figure 5). Thus, low sensitivity but also less demanding detection techniques (i.e., cytomorphology) might be used upfront with the methods currently in use for detecting the M-component and the diagnosis of the most aggressive plasma cell disease category (PCL). In turn, more sensitive determinations would be reserved for patients suspected of having a plasma cell neoplasm with negative cytomorphologic results in blood. Most patients with disseminated disease (e.g., MGUS, SMM and MM) will potentially show CTPC by NGF, supporting the diagnosis of a PC neoplasm. In addition, high CTPC counts in blood is highly suggestive of a MM (vs. MGUS), based on the high specificity (approximately 80%) of the next-generation flow assay; in such cases, BM examination is strongly recommended for a final diagnosis (Figure 5). In contrast, in a significant fraction of MGUS cases, no CTPC are detected, which might be of great utility for avoiding or delaying the BM study. In turn, cases with low CTPC counts in blood will most frequently be associated with MGUS, low-risk SMM and MGUS-like MM (Figure 5). In such cases, planning for a BM analysis might be considered depending on the presence vs. absence of symptoms and the amount of the serum monoclonal component (Figure 5) [21]. Similarly, the presence of CTPC in the blood of treated MM patients can be viewed as a surrogate marker of persistence of BM disease, and thereby used for more efficient planning of BM sampling.

## 6. Concluding Remarks

In recent years, new approaches have been developed that allow for the highly-sensitive detection of CTPC in patients with plasma cell neoplasms. From a clinical point of view, the detection of CTPC provides useful and relevant information for the differential diagnosis and prognostic stratification of patients with plasma cell neoplasms at diagnosis. At the same time, it enables more frequent, minimally invasive monitoring of both newly-diagnosed MGUS and treated MM patients. Overall, the presence of CTPC is associated with disseminated disease at diagnosis, as well as a higher risk of malignant transformation of MGUS and a poorer outcome (i.e., decreased progression free survival and/or overall survival rates) in both newly-diagnosed and treated MM. Based on all the above, we envisage that the currently available highly-sensitive CTPC techniques such as NGF or NGS will be soon incorporated into routine laboratory diagnostics for the diagnostic work-up and monitoring of newly-diagnosed and treated plasma cell neoplasms. Although both techniques are complementary, they hold similar levels of sensitivity with a comparable prognostic impact. Thus, NGF in combination with serum immunofixation might be preferred due to its higher applicability and broader availability without the need for a diagnostic sample.

## Figures and Tables

**Table 1:** Immunophenotypic profile of normal PC populations in secondary lymphoid tissues (e.g., tonsils), blood and bone marrow.

Type of Marker and Function	Target Molecule	Tonsil PC	Blood PC	Bone Marrow PC	References
**Activation markers**	CD71	Low	−	−	[39,52,53,54,55,56,57,58,59,60,61,62,63,64,65,66,67,68,69,70,71,72,73]
	CD37	Low	−	−	
	CD39	+	+	−	
	CD45RB	+	−/+	−	
	CD52	Low	NT	−	
	CD53	+	−	−	
	CD45	+	Low	−/+	
	CD45RO	−	−	−/+	
	CD45RA	+	+	−/+	
	CD200	−	−	−/Low	
	CD10	Low	−/Low	−/Low	
	CD28	−/Low	−/Low	−/Low	
	CD9	+	−/Low	+	
	CD43	+	+	+	
	CD361	++	++	++	
	CD38	+	+	++	
	CD27	Low	+	++	
	CD63	+	+	Low	
**Adhesion molecules**	CD100	Low	−	−	[39,52,53,54,57,60,61,62,63,64,66,70,72,74,75,76,77,78,79,80,81,82,83,84]
	CD18	Low	−	−	
	CD62L	−	−/+	−	
	CD47	+	−	−	
	CD11a (LFA-1)	+	−/Low	−/+	
	CD56 (NCAM)	−	−	−/+	
	CD49e (VLA-5)	−	−	−/+	
	CD99	+	+	+	
	CD44 (HCAM)	+	+	+	
	CD50 (ICAM-3)	+	−/Low	+	
	CD49f (ITGA6)	−	Low	+	
	CD98	+	+	+	
	CD54 (ICAM-1)	+	−/+	+	
	CD31	Low	+	++	
	CD106 (VCAM-1)	−	−	++	
	CD49d (VLA-4)	Low	+	++	
	CD97	Low	Low	Low	
	CD329 (SIGLEC 8)	Low	+	NT	
	CD363 (S1PR1)	−	−/+	−	
	CD82	+	+	+	
	CD81	+	+	+	
	CD362	−/+	++	+	
	CD138	−	−/+	+	
**BCR signalling molecules**	CD22	+	−/Low	−	[39,53,54,70,85,86]
	CD79b	Low	−	−	
	HLA-DR	++	−/+	−	
	CD19	+	Low	−/+	
	CD20	+	−/+	−/Low	
	CD21	+	−/+	−/Low	
	CD79a	−	−/+	+	
**Cell migration and chemokine receptors**	CD196 (CCR6)	−/+	−	−	[39,53,57,62,87,88]
	CD184 (CXCR4)	Low	−/+	−/+	
	CD185 (CXCR5)	−/+	−/+	−/Low	
**Complement receptors**	CD46	+	NT	−	[54,64,65,89,90,91,92]
	CD35	−/Low	−/Low	−/+	
	CD55	+	+	+	
	CD58	+	−	+	
	CD59	+	+	+	
**Co-stimulatory molecules**	CD72	Low	−	−	[39,54,62,73,93,94,95,96,97,98,99]
	CD80 (B7-1)	−	Low	−	
	CD40	+	Low	+	
	CD86 (B7-2)	Low	+	Low	
	CD272 (BTLA)	−/Low	+	Low	
	CD126 (IL-6Rα)	Low	+	−	
	CD130 (IL-6Rβ)	+	Low	Low	
	CD307a (FCRL-1)	−	Low	−	
	CD74	Low	−	−	
	CD305 (LAIR1)	−	−/+	−	
	CD32	Low	+	+	
	CD85j	Low	+	+	
	CD210a	−	−	Low	
**Receptors of the SLAM family**	CD84 (SLAMF5)	−/Low	−/+	−	[54,62,72,94,100]
	CD352 (SLAMF6)	++	++	+	
	CD150 (SLAMF1)	Low	+	+	
	CD48 (SLAMF2)	Low	++	+	
	CD229 (SLAMF3)	++	+	+	
	CD319 (SLAMF7)	−/+	−/+	+	
**PC survival-associated molecules**	CD357 (TNFRSF18)	−	Low	−	[39,53,54,62,72,73,94,96,101,102,103,104,105]
	CD257 (BAFF)	−/+	−/+	+	
	CD269 (BCMA)	−/Low	Low	+	
	CD268 (BAFF-R)	+	−	+	
	CD261 (TRAIL-R1)	−	−	+	
	CD358 (TNFSF21)	−	−	Low	
	CD270 (TNFRSF14)	Low	+	Low	
	CD262 (TRAIL-R2)	−	−	Low	
	Bcl-2	−	Low	+	
	CD274 (PD-L1)	+	+	−	
	CD95 (Fas-L)	Low	+	−	
**PC proliferation and Ig production**	Ki67	−/+	−/+	−/Low	[39,86,106,107]
	Vs38c	−/+	−/+	+	

Markers recurrently reported to be absent on normal PC populations include: CD1a, CD1b, CD1d, CD2, CD3, CD4, CD5, CD6, CD7, CD8, CD11b, CD11c, CD13, CD14, CD15, CD16, CD23, CD24, CD25, CD26, CD29, CD34, CD36, CD41, CD42a, CD42b, CD49a, CD49c, CD51, CD57, CD61, CD62E, CD62P, CD64, CD66a, CD66b, CD66c, CD66d, CD66e, CD68, CD69, CD70, CD73, CD83, CD85d, CD85k, CD87, CD88, CD89, CD90, CD91, CD93, CD96, CD117, CD152, CD170, CD244 (SLAMF4), CD258, CD264, CD267, CD275, CD283 (TLR3), CD289 (TLR9), CD307b-d, CD328, and CD354 (TREM-1). Expression profiles denoted above are based on conventional flow cytometry, immunofluorescence microscopy and/or immunohistochemistry. BCR, B-cell receptor; Ig, immunoglobulin; NT, not tested; PC, plasma cells; SLAM, signaling lymphocytic activation molecules.

**Table 2:** Advantages and disadvantages of the most frequently used methods for detection of circulating tumor plasma cells in patients diagnosed with plasma cell neoplasms.

	Cytology	IMF	MFC	NGF	ASO-qPCR	NGS
**Availability**	High	Low	High	High	Intermediate	Limited
**Applicability**	≈100%	≈100%	≈100%	≈100%	42% to75%	80–90%
**Sensitivity**	<10^−2^	<10^−4^	≤10^−4^	≤2 × 10^−6^	≤10^−5^–10^−6^	≤1 × 10^−6^
**Specificity**	Limited	Limited	High	High	High	High
**Standardized**	Yes	No	Ongoing	Yes	Yes	Ongoing
**Quantitative**	Yes (high counts)	Yes	Yes	Yes	Yes	Yes
**Diagnostic sample**	Not required	Not required	Not required	Not required	Mandatory	Mandatory
**Global sample analysis**	Yes	No	Yes	Yes	No	No
**Time to results**	<2 h	4 h	2–3 h	3–4 h	3–4 weeks	≥7 days
**Fresh sample**	Yes	Yes	Yes (<36 h)	Yes (<36 h)	No	No
Sample pre-treatment *	No	Yes	No	No	Yes	Yes
**Data analysis/interpretation**	Subjective	Slightly subjective	Slightly subjective	More objective	Slightly subjective	More objective
**CTPC detection principle**	DFN	Ig light-chain restriction	DFN and LAIP	DFN and LAIP	Patient-specific *IGH-V(D)J* gene rearrangements	Patient-specific *IGH-V(D)J* gene rearrangements ^¥^
**Additional biological characterization of CTPC**	No	No	Yes	Yes	No	Yes
**Prognostic factor in MGUS**	NT	Yes	NT	Yes	NT	NT
**Prognostic factor in SMM**	NT	Yes	Yes	Limited	NT	NT
**Prognostic factor in MM**	Yes	Yes	Yes	Yes	Yes	Yes
**Relative Cost**	Low	High	Intermediate	Intermediate	Intermediate	High

* Sample pre-treatment includes density gradient MNC- or magnetic/FACS- isolation. ^¥^ Including also potentially analysis of Ig light gene rearrangements. ASO-qPCR, allele-specific oligonucleotide quantitative real-time polymerase chain reaction; CTPC, circulating tumor plasma cells; DFN, different from normal; FACS, fluorescence activated cell sorting; Ig, immunoglobulin; IGH, Ig heavy chain; IMF, immuno-fluorescence microscopy; LAIP, leukemia associated immunophenotype; MGUS, monoclonal gammopathy of undetermined significance; MFC, multiparameter flow cytometry; MM, multiple myeloma; MNC, mononuclear cells; NGF, next generation flow; NGS, next generation sequencing; NT, not tested; SMM, smoldering MM.

**Table 3:** Prognostic impact of circulating tumor plasma cells on newly diagnosed and treated plasma cell neoplasms patients as assessed by distinct techniques.

Methodology	Diagnosis					Treated		
	MGUS	SMM		MM		MM		References
	TTP/PFS	TTP	OS	PFS	OS	PFS	OS	
**Cytology**	NT	NT	NT	NT	1.1 vs. 4.1y ^a^	NT	NT	[30,110]
**IMF**	138m vs. NR ^b^	12 vs. 57m ^c^	49 vs. 148m ^b^	NT	2.4 vs. 4.5y ^d^	6.2 vs. 22.5m ^e^	NT	[19,23,25,27,115]
**MFC**	NT	10m vs. NR ^b^	NT	25 vs. 43m ^b^ (TTNT *)	54 vs. 89m ^b^	15.1m vs. 29.6m ^b^	41m vs. NR ^b^	[22,26]
**NGF**	31m vs. NR ^f^	25% vs. 0% at 2y (*p >* 0.05) ^g^	NT	22m vs. NR ^g^	67% vs. 0% at 2y ^g^	9 vs. 46m ^b^	NT	[21,35]
**ASO-qPCR**	NT	NT	NT	26 vs. 66m ^b^	53 vs. 66m (*p* > 0.05) ^b^	4 vs. 15m ^b^	17 vs. 52m ^b^	[118]
**NGS**	NT	NT	NT	22.6 vs. 47.5m^h^ 26.7 vs. 41.3m ^i^	>55m ^h,i^	NT	NT	[119]

* TTNT, defined as time from diagnosis to next therapy due to documented relapse or progression of disease. ASO-qPCR, allele-specific oligonucleotide quantitative real-time polymerase chain reaction; IMF, immunofluorescence microscopy; m, months; MFC, multiparametric flow cytometry; MGUS, monoclonal gammopathy of undetermined significance; MM, multiple myeloma; NGF, next generation flow; NGS, next generation sequencing; NR, not reached; NT, not tested; OS, overall survival; PFS, progression-free survival; SMM, smoldering MM; TTP, time to progression; y; years. ^a^ ≥5% vs. <5% CTPC; ^b^ CTPC+ vs. CTPC-; ^c^ >5000 vs. ≤5000 CTPC/µL; ^d^ ≥4% vs. <4% CTPC; ^e^ ≥0.2 × 10^6^ vs. <0.2 × 10^6^ CTPC/L; ^f^ ≥0.058 vs. <0.058 CTPC/µL; ^g^ ≥0.1 vs. <0.1 CTPC/μL; ^h^ high vs. low expression levels of the *CENPF* gene; ^i^ high vs. low expression levels of the *LGALS1* gene.
